# Association of mismatch repair status with survival and response to neoadjuvant chemo(radio)therapy in rectal cancer

**DOI:** 10.1038/s41698-020-00132-5

**Published:** 2020-09-07

**Authors:** Shu-Biao Ye, Yi-Kan Cheng, Lin Zhang, Yi-Feng Zou, Ping Chen, Yan-Hong Deng, Yan Huang, Jian-Hong Peng, Xiao-Jian Wu, Ping Lan

**Affiliations:** 1 Department of Colorectal Surgery, The Sixth Affiliated Hospital, Sun Yat-sen University, Guangdong Institute of Gastroenterology, Guangdong Provincial Key Laboratory of Colorectal and Pelvic Floor Diseases, Guangzhou, Guangdong People’s Republic of China; 2 Department of Radiation Oncology, The Sixth Affiliated Hospital, Sun Yat-sen University, Guangdong Institute of Gastroenterology, Guangdong Provincial Key Laboratory of Colorectal and Pelvic Floor Diseases, Guangzhou, People’s Republic of China; 3 State Key Laboratory of Oncology in South China, Collaborative Innovation Centre for Cancer Medicine, Sun Yat-sen University Cancer Center, Guangzhou, People’s Republic of China; 4 Department of Clinical Laboratory, Sun Yat-sen University Cancer Center, Guangzhou, People’s Republic of China; 5 Department of VIP Region, Sun Yat-sen University Cancer Center, Guangzhou, People’s Republic of China; 6 Department of Oncology, The Sixth Affiliated Hospital, Sun Yat-sen University, Guangdong Institute of Gastroenterology, Guangdong Provincial Key Laboratory of Colorectal and Pelvic Floor Diseases, Guangzhou, People’s Republic of China; 7 Department of Pathology, The Sixth Affiliated Hospital, Sun Yat-sen University, Guangdong Institute of Gastroenterology, Guangdong Provincial Key Laboratory of Colorectal and Pelvic Floor Diseases, Guangzhou, People’s Republic of China; 8 Department of Colorectal Surgery, Sun Yat-sen University Cancer Center, Guangzhou, People’s Republic of China

**Keywords:** Rectal cancer, Predictive markers, Radiotherapy

## Abstract

Prior reports have indicated that defective mismatch repair (MMR) has a favorable impact on outcome in colorectal cancer patients treated with surgery, immunotherapy, or adjuvant chemotherapy. However, the impact of MMR status on response to neoadjuvant radiotherapy in rectal cancer is not well understood. Here we report that dMMR was associated with improved disease-free survival (DFS) (*P* = 0.034) in patients receiving neoadjuvant chemotherapy (NCT). Patients with dMMR tumors who received neoadjuvant chemoradiotherapy (NCRT) achieved significantly worse DFS (*P* = 0.026) than those treated with NCT. Conversely, NCRT improved DFS (*P* = 0.043) in patients with pMMR tumors, especially for stage III disease with improved DFS (*P* = 0.02). The presence of dMMR was associated with better prognosis in rectal cancer patients treated with NCT. NCT benefited patients with dMMR tumors; while NCRT benefited patients with stage III disease and pMMR tumors. Patients stratified by MMR status may provide a more tailored approach to rectal cancer neoadjuvant therapy.

## Introduction

Deficient mismatch repair (dMMR) or microsatellite instability (MSI) is one of the well-established molecular biomarkers in colorectal cancer (CRC) and MSI testing has been recommended for all CRC patients according to the National Comprehensive Cancer Network (NCCN) guidelines^1^ due to its aid in dictating management^2,3^. For instance, early-stage colorectal adenocarcinomas testing positive for MSI may carry a favorable prognosis and therefore do not require adjuvant chemotherapy^3–5^. Some studies suggest that these tumors are more prone to be sensitive to 5-FU chemo(radio)therapy, although this finding is controversial^6–10^. Even if a significantly decreased likelihood of pathologic complete response (pCR) has been demonstrated for dMMR patients after chemoradiation^6^ in the largest sample-sized study, no study has been reported to investigate the association of MMR status with survival and response to neoadjuvant radiotherapy (NRT) with a comparison of patients receiving neoadjuvant chemotherapy (NCT) with or without radiotherapy.

Neoadjuvant chemoradiotherapy (NCRT) and surgery plus adjuvant chemotherapy is recommended for stage II/III rectal cancer patients^1^. Radiotherapy is a crucial component of neoadjuvant care, which has been demonstrated to improve local recurrence-free survival (LRFS) but not overall survival^11,12^ and to be associated with higher rates of related adverse events^13^. Thus, the exploration of different avenues of neoadjuvant therapy, such as the omission of NRT or selective NRT before chemotherapy and total mesorectal excision, is currently under investigation^14,15^ (PROSPECT clinical trial, etc.). However, for now, these patients receiving NRT or not are determined on an individual basis, and there is a tremendous demand for predictive biomarkers to select optimal patients who can benefit the most from NRT. Although immunotherapy alone or combined with conventional therapy are being rapidly developed for dMMR CRCs^16^, the predictive value of MMR status in NRT remains undefined in rectal cancer. We therefore aimed to investigate the association of MMR status with survival and response to neoadjuvant chemo(radio)therapy.

The new attempts of selective NRT or the omission of NRT that have recently emerged in our center (NCT01211210, NCT02217020, etc.)^17,18^ have provided a direct comparison between NCRT and NCT, which is ideal for determining the impact of MMR status on response to NRT. This is the first study to utilize multicenter data to investigate this issue in locally advanced rectal cancer.

## Results

### Patient characteristics and associations with MMR status

Patients with dMMR tumors (*n* = 66) were significantly younger than patients with mismatch repair-proficient (pMMR) tumors (*n* = 949) (Table 1, Supplementary Table 1). NCRT was given in 591 patients (58.2%), whereas 424 patients (41.8%) received NCT. The preoperative radiation dose exhibited very minor variability, with 95.3% receiving 45–50.4 Gy over 5 weeks.

**Table 1:** Comparative baseline characteristics for mismatch repair status.

Characteristics	MMR-proficient, *n* = 949	MMR-deficient, *n* = 66	*P* value^#^
Sex (%)			0.683
Male	653 (68.8)	47 (71.2)	
Age (year, %)			<0.001^*^
≤60	570 (60.0)	54 (81.8)	
>60	379 (40.0)	12 (18.2)	
Comorbidity (%)			0.369
Yes	218 (23.0)	12 (18.2)	
No	731 (77.0)	54 (81.8)	
BMI (%)			0.234
Mean (SD)	22.5 (3.2)	22.0 (3.0)	
Clinical T stage (%)			0.249
T1	1 (0.0)	0 (0)	
T2	15 (1.6)	1 (1.5)	
T3	717 (75.6)	44 (66.7)	
T4	216 (22.8)	21 (31.8)	
Clinical N stage (%)			0.783
N0	207 (21.8)	16 (24.2)	
N1	473 (49.8)	30 (45.5)	
N2	269 (28.3)	20 (30.3)	
Distance from anal verge (%)			0.126
≤5 cm	442 (46.6)	38 (57.6)	
>5 and ≤10 cm	453 (47.7)	23 (34.8)	
>10 cm	54 (65.7	5 (7.6)	
ypStage (%)			0.137
0–I	318 (33.5)	16 (24.2)	
II–III	631 (66.5)	50 (75.8)	
pCR (%)	60 (6.3)	6 (9.1)	0.387
Differentiation (%)			0.090
Well	167 (17.6)	17 (25.8)	
Moderately	652 (68.7)	36 (54.5)	
Poorly	105 (11.1)	11 (16.7)	
NS	25 (2.6)	2 (3.0)	
Neoadjuvant radiotherapy (NRT) (%)			0.090
Yes	546 (57.5)	45 (68.2)	
No	403 (42.5)	21 (31.8)	
Adjuvant chemotherapy (ACT) (%)			0.651
Yes	815 (85.9)	58 (87.9)	
No	134 (14.1)	8 (12.1)	
Radiotherapy dose, Gy (%)			0.08
0	403 (42.5)	21 (31.8)	
<45	25 (2.6)	4 (6.1)	
≥45 and <50.4	521 (54.9)	41 (62.1)	

*MMR* mismatch repair, *SD* standard deviation, *BMI* body mass index, *NRT* neoadjuvant radiotherapy, *pCR* pathologic complete response, *NS* not sure.

^#^Clinicopathological differences between the pMMR and dMMR groups were compared with the Mann–Whitney *U* test for continuous variables and *χ*^2^ test (or Fisher’s exact test, if appropriate) for categorical data.

*Statistically significant.

For patients with dMMR tumors, although the percentage of stage III patients in NCT group was higher than NCRT group (90.5% vs. 68.9%, *P* = 0.07), downstaging rate was also higher than NCRT group (81.0% vs. 62.2%, *P* = 0.163) (Table 2). Only 1 patient (4.8%) received single agent chemotherapy in NCT group, while 11 patients (24.4%) received single agent chemotherapy (*P* = 0.001) in NCRT group (Table 3), of whom 7 patients (63.6%) had disease recurrence. As for patients with pMMR tumors, compared to the NCT group, patients in the NCRT group were more likely to have T3/T4 tumors (*P* = 0.006), and poorly differentiated (*P* < 0.001) and distally located tumors (*P* = 0.001). Nevertheless, the rate of downstaging and pCR were higher (*P* < 0.001, *P* = 0.002) and the rate of advanced pathologic stage was lower (*P* = 0.0017) in NCRT group (Table 2). Such trends were also demonstrated in rectal cancer with pMMR tumors and stage III disease (Supplementary Table 2).

**Table 2:** Baseline characteristics.

Characteristics	MMR-proficient (*n* = 949)			MMR-deficient (*n* = 66)		
	NCT (*n* = 403)	NCRT (*n* = 546)	*P* value^#^	NCT (*n* = 21)	NCRT (*n* = 45)	*P* value^#^
Age (year, %)			0.538			0.746
≤60	244 (60.5)	326 (59.7)		18 (85.7)	36 (80.0)	
>60	159 (39.5)	220 (40.3)		3 (14.3)	9 (20.0)	
Sex (%)			0.419			0.771
Male	283 (70.2)	370 (67.8)		14 (66.7)	33 (73.3)	
BMI			0.574			0.741
Mean (SD)	22.4 (3.4)	22.5 (3.0)		21.8 (2.8)	22.0 (3.1)	
Comorbidity (%)			0.806			0.738
Yes	91 (22.6)	127 (23.3)		3 (14.3)	9 (20)	
Clinical T stage (%)			0.006^*^			0.356
T1	1 (0.2)	0 (0)		0 (0)	0 (0)	
T2	6 (1.5)	9 (1.6)		1 (4.8)	0 (0)	
T3	324 (80.4)	393 (72.0)		13 (61.9)	31 (68.9)	
T4	72 (17.9)	144 (26.4)		7 (33.3)	14 (31.1)	
Clinical N stage (%)			0.058			0.089
N0	100 (24.8)	107 (19.6)		2 (9.5)	14 (31.1)	
N1	184 (45.7)	289 (52.9)		13 (61.9)	17 (37.8)	
N2	119 (29.5)	150 (27.5)		6 (28.6)	14 (31.1)	
Clinical stage (%)			0.039^*^			0.07
II	100 (24.8)	107 (19.6)		2 (9.5)	14 (31.1)	
III	303 (75.2)	439 (80.4)		19 (90.5)	31 (68.9)	
Distance from anal verge (%)			<0.001^*^			0.395
≤5 cm	164 (40.7)	278 (50.9)		10 (47.6)	28 (62.2)	
>5 and ≤10 cm	198 (49.1)	255 (46.7)		10 (47.6)	13 (28.9)	
>10 cm	41 (10.2)	13 (2.4)		1 (4.8)	4 (8.9)	
ypStage (%)			0.018^*^			0.759
0–I	118 (29.3)	200 (36.6)		6 (28.6)	10 (22.2)	
II–III	285 (70.7)	346 (63.4)		15 (71.4)	35 (77.8)	
pCR (%)	14 (3.5)	46 (8.4)	0.002^*^	2 (10.0)	4 (9.0)	0.639
Downstaging status (%)			0.003^*^			0.163
Downstaged	266 (66.0)	409 (74.9)		17 (81.0)	28 (62.2)	
Same or higher	137 (34.0)	137 (25.1)		4 (19.0)	17 (37.8)	
NAR score			<0.001^*^			0.53
Mean (SD)	21.8 (28.3)	18.1 (26.4)		14.8 (8.6)	32.7 (44.4)	
Differentiation (%)			<0.001^*^			0.426
Well	93 (23.1)	74 (13.6)		8 (38.1)	9 (20.0)	
Moderately	265 (65.8)	387 (70.9)		10 (47.6)	26 (57.8)	
Poorly	40 (9.9)	65 (11.9)		3 (14.3)	8 (17.8)	
NS	5 (1.2)	20 (3.7)		0 (0)	2 (4.4)	

*MMR* mismatch repair, *SD* standard deviation, *BMI* body mass index, *NCT* neoadjuvant chemotherapy, *NCRT* neoadjuvant chemoradiotherapy, *pCR* pathologic complete response, *NAR score* neoadjuvant rectal score, *NS* not sure, *5FU* 5-fluorouracil, *FOLFOX* 5-fluorouracil+oxaliplatin, *XELOX* xeloda+ oxaliplatin, *FOLFOXIRI* 5-fluorouracil+oxaliplatin+irinotecan.

^#^Clinicopathological differences between the pMMR and dMMR groups stratified by neoadjuvant treatment groups were compared with the Mann–Whitney *U* test for continuous variables and *χ*^2^ test (or Fisher’s exact test, if appropriate) for categorical data.

*Statistically significant.

**Table 3:** Treatment characteristics.

Characteristics	MMR-proficient (*n* = 949)			MMR-deficient (*n* = 66)		
	NCT (*n* = 403)	NCRT (*n* = 546)	*P* value^#^	NCT (*n* = 21)	NCRT (*n* = 45)	*P* value^#^
Neoadjuvant chemotherapy regimen (%)			<0.001^*^			0.001^*^
5FU/Xeloda	5 (1.2)	163 (29.9)		1 (4.8)	11 (24.4)	
FOLFOX/XELOX	314 (77.9)	360 (65.9)		15 (71.4)	34 (75.6)	
FOLFOXIRI	83 (20.6)	0 (0)		5 (23.8)	0 (0)	
Others	1 (0.2)	9 (1.6)		0 (0)	0 (0)	
NS	0 (0)	14 (2.6)		0 (0)	0 (0)	
Adjuvant chemotherapy (%)			0.093			0.702
Yes	355 (88.1)	460 (84.2)		18 (85.7)	40 (88.9)	
No	48 (11.9)	86 (15.8)		3 (14.3)	5 (11.1)	

*5FU* 5-fluorouracil, *FOLFOX* 5-fluorouracil+oxaliplatin, *XELOX* xeloda+ oxaliplatin, *FOLFOXIRI* 5-fluorouracil+oxaliplatin+irinotecan, *NS* not sure.

^#^Clinicopathological differences between the pMMR and dMMR groups stratified by neoadjuvant treatment groups were compared with the Mann–Whitney *U* test for continuous variables and *χ*2 test (or Fisher’s exact test, if appropriate) for categorical data.

### Association between MMR status and survival

**Fig. 1:**
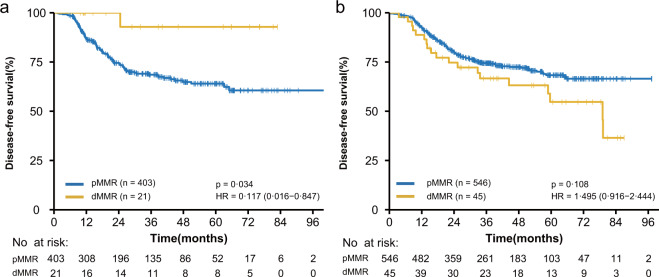
Association between MMR status and disease-free survival (DFS) according to neoadjuvant treatment. **a** DFS in patients with neoadjuvant chemotherapy alone (NCT) by DNA mismatch repair (MMR) status. **b** DFS in patients with neoadjuvant chemoradiotherapy (NCRT) by MMR. dMMR-deficient mismatch repair; pMMR-proficient mismatch repair. HR hazard ratio, CI confidential interval.

Although MMR status was not a prognostic biomarker in the whole cohort even after propensity match analysis (Supplementary Fig. 1), dMMR was associated with improved DFS (HR, 0.117; 95% CI, 0.016–0.847; *P* = 0.034, Fig. 1, Table 4) in NCT group, whereas no significant correlation between MMR status and DFS (HR, 1.495; 95% CI, 0.916–2.444; *P* = 0.108, Fig. 1) was shown in the NCRT group (Table 4).

**Table 4:** DFS by MMR and neoadjuvant radiotherapy status in univariable and multivariable analysis adjusted for clinical characteristics.

MMR and NRT status	No. of patients (*n* = 1015)	5-year rate (%)	Univariable	Multivariable		
			*P* value^a^	HR	95% CI	*P* value^b^
NCRT	591					
dMMR	45	41	0.070	1.495	0.916–2.444	0.108
pMMR	546	66				
NCT	424					
dMMR	21	92	0.020^*^	0.117	0.016–0.847	0.034^*^
pMMR	403	59				
dMMR	66					
NCRT	45	41	0.036^*^	10.580	1.333–83.974	0.026^*^
NCT	21	92				
pMMR	949					
NCRT	546	66	0.033^*^	0.763	0.587–0.991	0.043*
NCT	403	59				

*DFS* disease-free survival, *dMMR* deficient mismatch repair, *pMMR* proficient mismatch repair, *NRT* neoadjuvant radiotherapy, *NCT* neoadjuvant chemotherapy, *NCRT* neoadjuvant chemoradiotherapy, *HR* hazard ratio, *CI* confidential interval.

*Statistically significant.

^a^Univariable survival between these subgroups was compared by using the log-rank test.

^b^Cox proportional-hazards regression analysis, which adjusted for clinicopathologic covariates (including age, gender, clinical T or N stage, localization, and neoadjuvant radiotherapy) were used to calculate *P* values.

### Association between neoadjuvant treatment and survival

**Fig. 2:**
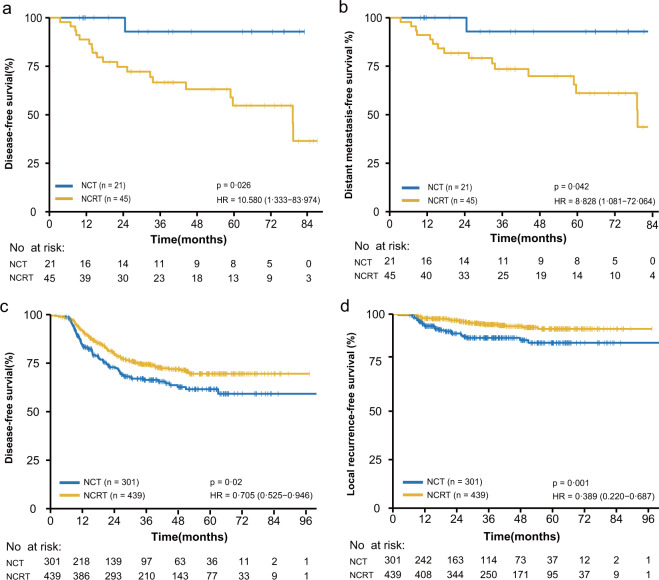
Association between neoadjuvant treatment and survival according to mismatch repair (MMR) status. **a** Disease-free survival (DFS), and **b** distant metastasis-free survival (DMFS) in patients with deficient MMR (dMMR) by neoadjuvant radiotherapy. **c** DFS and **d** Local recurrence-free survival (LRFS) in patients with stage III disease and proficient MMR (pMMR) tumors by neoadjuvant radiotherapy. NCT neoadjuvant chemotherapy, NCRT neoadjuvant chemoradiotherapy, HR hazard ratio, CI confidential interval.

Compared to NCT, NCRT was associated with a worse DFS (HR, 10.580; 95% CI, 1.333–83.974; *P* = 0.026) and DMFS (HR, 8.828; 95% CI, 1.081–72.064; *P* = 0.042) in rectal cancer patients with dMMR tumors (Fig. 2, Table 5) but was correlated with an improved DFS (HR, 0.763; 95% CI, 0.587–0.991; *P* = 0.043) and LRFS (HR, 0.403; 95% CI, 0.241–0.673; *P* = 0.001) in pMMR patients (Supplementary Fig. 2, Supplementary Tables 3 and 4). For patients with stage III disease and pMMR tumors, the addition of NRT could achieve better DFS (HR, 0.705; 95% CI, 0.525–0.946; *P* = 0.02) and LRFS (HR, 0.389; 95% CI, 0.220–0.687; *P* = 0.001) (Fig. 2, Table 6). Competing risk estimate also showed the similar trends in patients with dMMR tumors, pMMR or pMMR with stage III disease (Supplementary Fig. 3). No benefit from the addition of NRT was shown in patients with stage II disease and pMMR tumors. However, compared to T3N0 disease, T4N0 disease was associated with worse LRFS in these subsets of patients receiving NCT (HR, 8.108; 95% CI, 1.798–36.559; *P* = 0.006), whereas there was no correlation between T disease and LRFS in NCRT group (Supplementary Fig. 4).

**Table 5:** Survival by neoadjuvant radiotherapy for dMMR patients in univariate and multivariate analysis adjusted for clinical characteristics.

	DFS		DMFS	
	Hazard ratio (95% CI)	*P* value^#^	Hazard ratio (95% CI)	*P* value^#^
*Univariate*				
NRT				
With vs. without	8.657 (1.158–64.700)	0.010^*^	6.871 (0.146–51.870)	0.030^*^
*Multivariate*				
NRT				
With vs. without	10.580 (1.333–83.974)	0.026^*^	8.828 (1.081–72.064)	0.042^*^
Age				
>60 yr vs. ≤60 yr	0.844 (0.215–3.309)	0.808	1.001 (0.995–4.2556)	0.994
Sex				
Female vs. male	1.102 (0.394–3.081)	0.853	1.300 (0.447–3.778)	0.630
Clinical T stage				
T2–3 vs. T4	2.702 (0.967–7.550)	0.058	2.904 (0.934–9.032)	0.065
Clinical N stage				
N0	1.000		1.000	
N1	1.112 (0.361–3.422)	0.855	1.427 (0.427–4.762)	0.563
N2	3.133 (0.882–11.133)	0.077	3.837 (0.917–16.052)	0.066
Localization				
Low	1.000		1.000	
Middle	0.295 (0.076–1.141)	0.077	0.160 (0.029–0.888)	0.036^*^
High	0.212 (0.023–1.945)	0.170	0.226 (0.023–1.963)	0.172

Among 66 patients with dMMR tumors, there were only four local recurrence events; thus, data of local recurrence-free survival were not shown in Table 5.

*dMMR* deficient mismatch repair, *DFS* disease-free survival, *DMFS* distant metastasis-free survival, *NRT* neoadjuvant radiotherapy, *CI* confidential interval.

^#^Cox proportional-hazards regression analysis was used to calculate *P* values.

^*^Statistically significant.

**Table 6:** Survival by neoadjuvant radiotherapy for stage III pMMR patients in univariable and multivariable analysis adjusted for clinical characteristics.

	DFS		LRFS		DMFS	
	Hazard ratio (95% CI)	*P* value^#^	Hazard Ratio (95% CI)	*P* value^#^	Hazard Ratio (95% CI)	*P* value^#^
*Univariate*						
NRT						
With vs. without	0.695 (0.525–0.922)	0.010^*^	0.426 (0.250–0.727)	0.002	0.778 (0.570–1.061)	0.100
*Multivariate*						
NRT						
With vs. without	0.705 (0.525–0.946)	0.020^*^	0.389 (0.220–0.687)	0.001^*^	0.793 (0.573–1.096)	0.158
Age						
>60 yr vs. ≤60 yr	0.990 (0.741–1.325)	0.950	1.060 (0.607–1.848)	0.839	0.904 (0.655–1.247)	0.538
Sex						
Female vs. male	1.005 (0.743–1.360)	0.975	0.571 (0.300–1.089)	0.089	1.200 (0.868–1.658)	0.270
Clinical T stage						
T2–3 vs. T4	1.442 (0.884–1.714)	0.219	2.078 (1.147–3.767)	0.019^*^	1.111 (0.769–1.605)	0.576
Clinical N stage						
N1 vs. N2	1.442 (1.081–1.924)	0.013^*^	2.216 (1.279–3.839)	0.005^*^	1.454 (1.061–1.994)	0.019^*^
Localization						
Low	1.000		1.000		1.000	
Middle	0.873 (0.649–1.175)	0.372	0.425 (0.234–0.774)	0.005^*^	1.017 (0.734–1.408)	0.920
High	1.390 (0.809–2.390)	0.233	0.922 (0.382–2.228)	0.858	1.288 (0.686–2.417)	0.431

*pMMR* proficient mismatch repair, *DFS* disease-free survival, *LRFS* local recurrence-free survival, *DMFS* distant metastasis-free survival, *NRT* neoadjuvant radiotherapy, *CI* confidential interval.

^#^Cox proportional-hazards regression analysis was used to calculate *P* values.

*Statistically significant.

### Association of treatment and survival in cohort without pCR

Since the reasons for MMR testing were not provided, and testing may have been performed in patients who did not respond well to neoadjuvant therapy and MMR status could not be determined on the surgical pathological specimen for patients with pCR, we reanalyzed the association of MMR status with survival and response to neoadjuvant therapy for remaining patients excluding those with pCR.

Baseline clinical characteristics of the remaining patients excluding those with pCR are shown in Supplementary Table 5. Compared to NCT, NCRT was associated with a significantly worse DFS (HR, 11.113; 95% CI: 1.395–88.520, *P* = 0.023) and DMFS (HR: 9.296, 95% CI: 1.123–76.934, *P* = 0.039) in patients with dMMR (Supplementary Table 6, Supplementary Fig. 4) but with an improved LRFS (HR: 0.438, 95% CI: 0.257–0.741, *P* = 0.002) in patients with pMMR tumors (Supplementary Table 7). In the remaining data, for patients with pMMR tumors and stage III disease, the omission of NRT could achieve worse DFS (HR, 0.731; 95% CI: 0.543–0.985, *P* = 0.039) and LRFS (HR: 0.423, 95% CI: 0.237–0.754, *P* = 0.004) (Supplementary Table 8, Supplementary Fig. 5). Although no benefit from the addition of NRT was shown in patients with stage II disease and pMMR tumors, the univariate analysis indicated that compared with T3 disease, T4 disease was associated with worse LRFS in these subsets of patients (HR, 5.403; 95% CI, 1.051–27.786; *P* = 0.044), whereas there was no correlation between T disease and LRFS in NCRT group (Supplementary Fig. 6).

## Discussion

In locally advanced rectal cancer, NRT is routinely used for improving local control^12^. Clinical trials have so far failed to increase disease-free and overall survival^11,12^. A pilot study from Memorial Sloan Kettering Cancer Centre has reported the potential feasibility of NCT without routine use of radiation therapy^19^ and thus the omission of radiotherapy in the preoperative treatment settings is under investigation^14^. Although numerous studies have demonstrated the impact of MMR status on the response to NCRT^6,10,19–22^, little is known about how MMR influences the outcome in patients with or without radiotherapy in neoadjuvant treatment. Therefore, the association between MMR status and the outcome of radio-responsiveness has not been well explored. Accordingly, in this large multicenter retrospective study that, to our knowledge, is the first reported to date, we have demonstrated that MMR status could serve as a valuable predictor to select optimal patients who benefit the most from NRT.

The dMMR patients in our cohort were significantly younger than pMMR patients because at least some of them had Lynch syndrome, similarly to patients in a recent published study^22^. Similar to colon cancer, dMMR is a significant prognostic factor in rectal cancer patients receiving NCT. Neoadjuvant (chemo)radiotherapy for rectal cancer with dMMR tumors seems to be radiosensitive^19^. However, their study only included 29 locally advanced rectal cancers and had no comparison with pMMR patients. Furthermore, Cercek et al. recently reported chemotherapy resistance for dMMR tumors^23^, which was inconsistent with our findings. The main reasons for the distinction might be that a portion of patients had metastatic disease and a majority of patients underwent NCRT after receiving initial NCT in their study. A recent study has evaluated the largest series of locally advanced rectal cancer patients with preoperative chemoradiotherapy (5086 patients) from the National Cancer Database (NCDB)^6^, including 4450 patients with pMMR tumors and 636 patients with dMMR tumors. After propensity matched and case-control analysis, dMMR was independently correlated with a reduced pCR rate on multivariable analysis, which indicated the potential chemo/radio-resistant role of dMMR in rectal cancer. Additionally, a poorer prognosis of rectal cancer patients with dMMR tumors has also been reported^8^. None of these studies has directly compared patients receiving neoadjuvant treatment with and without radiotherapy to determine the association of MMR status and the response to NRT. Our study first showed that NCT had a better survival outcome than NCRT in dMMR patients, which was consistent with the abovementioned notion of radio(chemo)-resistance for dMMR tumors in the neoadjuvant setting^24^. One possible explanation involves the different chemotherapy regimens (mFOLFOX6 for FOWARC clinical trial and 4–6 cycles of mFOLFOXIRI for FORTUNE clinical trial, respectively) between NCRT and NCT group. The lower relapse rate mainly from distant control in NCT group may benefit from double or triple-agents chemotherapy regimen that the vast majority of patients (95.2%) received. Although substantial evidence showed dMMR colon cancers do not benefit from adjuvant FU/leucovorin^3,4^, preliminary data indicates that the addition of either oxaliplatin or irinotecan to FU/leucovorin may overcome this resistance in patients with dMMR^25,26^. Moreover, patients with dMMR tumors underwent single-agent NCRT had strikingly high disease recurrence rate (63.6%), which was consistent with the evidence that dMMR tumors had resistance to single-agent chemotherapy. Another possible explanation may be the association between the well-known frequent occurrence of BRAF mutations in dMMR tumors^27^ and resistance to NCRT^28^.

These findings raise the attention of clinicians to test MMR status in biopsy specimens, which may guide subsequent treatment. Such information could be quite valuable for the exploration of neoadjuvant treatment, for instance, the omission of radiotherapy in preoperative treatment settings or selective preoperative radiation^14,15^. Presuming that such resistance to NRT exists in dMMR rectal cancer patients, immunotherapy may provide a pathway to help navigate this situation and has been shown as an alternative to conditional therapy in three cases in our center. A 27 years old male patient and a 61 years old female patient were diagnosed with cT4bN2M0 dMMR rectal cancer, both of whom were case-reported recently^29^. The first patient received six cycles of nivolumab and total mesorectal excision and the pathologic examination result showed complete response. After the second patient received four cycles of FOFOLXIRI, the primary tumor shrank. Then after eight cycles of nivolumab, she was diagnosed with a clinical complete response (cCR) by comprehensive examination. The third male patient (Supplementary case report), 35 years, with dMMR tumor was diagnosed with poor differentiate rectal adenoma carcinoma invading peritoneal reflection (cT4a-bN1bM0) in January 2018. After chemoradiotherapy, he had progression of disease with clinical stage cT4BN2M1 with lung and retroperitoneal lymph node metastases. After 16 cycles of anti-PD-1 immunotherapy, primary disease and lung metastases were invisible and retroperitoneal lymph nodes shrank a lot from the examination of PET/CT in August 20, 2019. Since a subset of dMMR patients has Lynch syndrome who tends to be young, omission of radiotherapy can preserve fertility function. Trial of immunotherapy in dMMR locally advanced rectal cancers is preparing in our center.

For rectal cancer patients with pMMR tumors, although more T3/T4 tumors, poor differentiation and distally located tumors were present in the NCRT group, they still achieved higher rates of pCR, lower neoadjuvant rectal (NAR) score and early ypStage. More importantly, this subset of patients had a significantly improved survival compared with that of NCT group. Such a trend has also been demonstrated in patients with pMMR tumors and stage III disease. ypStage and pCR following neoadjuvant therapy are well-known strong indicators of survival in rectal cancer^30–32^. Therefore, more patients with pCR and lower ypStage in NCRT group may yield a better treatment outcome. Although there was no correlation between NRT and survival in patients with pMMR tumors and stage II disease, T4 disease was significantly correlated with more local recurrence in the NCT alone group. Thus, our findings strongly recommended neoadjuvant radiation with fluorouracil-based chemotherapy for stage III and T4N0 diseases in rectal cancer patients with pMMR tumors, which is consistent with the NCCN guidelines^1^ and European Society for Medical Oncology (ESMO)^33^. Total neoadjuvant therapy (TNT), chemoradiotherapy and chemotherapy prior to resection, aims to reduce the risk of micrometastases and is increasingly used in practice. Moreover, clinical evidence and phase II clinical trials demonstrated that TNT improved the compliance rates and the incidence of pCR^34,35^. Therefore, TNT might be an alternative for pMMR patients to achieve higher pCR rate and improve survival.

While our study included the largest cohort of patients with stage II–III rectal cancer to explore the impact of MMR status on selecting patients who can benefit most from NRT and the findings are thought-provoking, these results remain limited by the selection bias inherent to any retrospective study and the lack of validation cohort. Thus, present investigation attempted to reduce bias via multivariable analysis and propensity matched analysis^36^ and lack of validation cohort was due to the recent exploration of selective radiation and rarity of dMMR in rectal cancer. Thus, further prospective, multicenter, and randomized studies are warranted to validate our findings. Although we only included stage II–III rectal cancer patients, the size of each group in dMMR cohort is still close to those reported by other institutions^8,37^. Moreover, the reasons for MMR testing were not provided, and MMR status could not be determined in surgical specimen for patients with pCR, which was perhaps the reason for the relatively low pCR rate (NCT may also lead to low pCR rate). Therefore, we reanalyzed the remaining patients, excluding those with pCR. Similar findings of the impact of MMR status on selective NRT were also shown in these subsets of patients. Finally, MMR testing was performed with IHC staining, which was not the recommended method of next generation sequencing (NSG)^38^ according to the latest NCCN guidelines.

In conclusion, this is the first comprehensive study to suggest that rectal cancer patients with dMMR tumors do not benefit from NRT, which indicates the probability of immunotherapy in neoadjuvant treatment settings for locally advanced rectal cancer patients with dMMR tumors; whereas improved survival may be seen in patients with pMMR tumors and stage III disease. Although the present study bridges the previous knowledge gaps, we would not advocate altering treatment decisions according to our findings unless it is validated by further prospective, multicenter, randomized, controlled clinical trials.

## Methods

### Patients

**Fig. 3:**
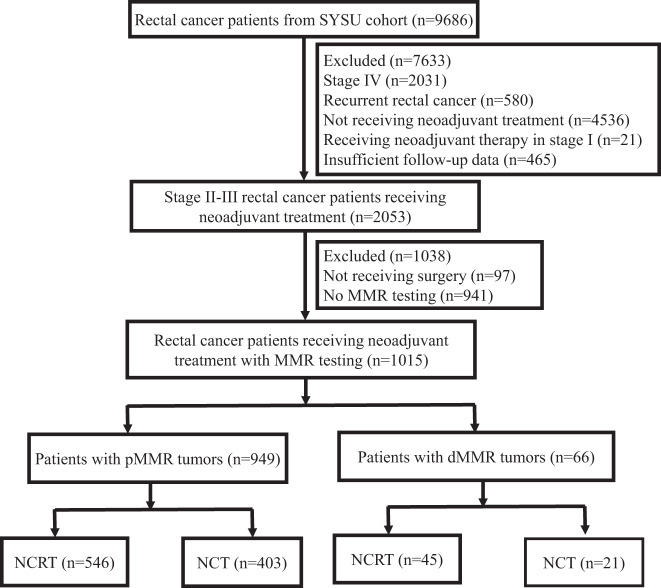
Flowchart depicting the process of patient inclusion. SYSU Sun Yat-sen University, MMR mismatch repair, pMMR proficient mismatch repair, dMMR deficient mismatch repair, NCRT neoadjuvant chemoradiotherapy, NCT neoadjuvant chemotherapy.

This retrospective multicenter study from Sun Yat-sen University (SYSU cohort) included 9686 patients who had histopathologically confirmed rectal adenocarcinoma and clinically confirmed locally advanced rectal cancer treated with neoadjuvant therapy in the Sixth Affiliated Hospital, Sun Yat-sen University and Sun Yat-sen University Cancer Center from December 2011 to September 2018. Only 1015 patients who had confirmed MMR status by tissue specimens were finally included. Locally advanced tumor was clinically confirmed disease with stage II (T3 to 4N0) or stage III (T1 to 4N1 to 2) with positive node defined as ≥1.0 cm in diameter on imaging. The inclusion criteria for patients receiving NCT were as follows: (1) patients from FOWARC clinical trial, (2) patients from FORTUNE clinical trial and (3) other patients underwent NCT according to physicians’ discretion and patients’ preference after the publications of FOWARC clinical trial^14,16^. A diagram depicting the selection process is outlined in Fig. 3. Clinicopathological features were extracted from prospectively maintained colorectal surgery databases (CSDs) and patient records, including clinical characteristics, pathologic features, neoadjuvant treatment, adjuvant treatment and oncologic outcomes. All patients signed informed consent before treatment. Our study was approved by the Ethics Committee of Sun Yat-sen University, the Sixth Affiliated Hospital (Ethics Approval Number: 2019ZSLYEC-176) and followed the reporting recommendation of tumor marker studies (REMARK) guidelines^39^.

### MMR status determination and analysis

All MMR status was performed by immunohistochemistry (IHC) for the proteins MLH1, MSH2, MSH6 and PMS2. The dMMR was defined as the loss of expression of one or more MMR proteins by IHC.

IHC staining for MMR proteins was performed on all specimens. ZSGB-Bio Solutions SPlink Detection Kits (Zhong Shan Jin Qiao, Beijing, China) and automated IHC/ISH slide staining instrument (The BenchMark XT platform) were used to stain the formalin-fixed, paraffin-embedded, 5-µm sections according to the manufacturer’s instructions. Staining was conducted with the use of diagnostic antibodies against MLH1 (clone ES05; Zhong Shan Jin Qiao, Beijing, China, 1:40), MSH2 (clone RED2; Zhong Shan Jin Qiao, Beijing, China, 1:200), MSH6 (clone UMAB258; Zhong Shan Jin Qiao, Beijing, China, 1:200) and PMS2 (clone EP51; Zhong Shan Jin Qiao, Beijing, China, 1:40). When nuclear staining was absent from all tumor cells but present in normal epithelial and stroma cells, protein expression was defined as absent (or abnormal).

### Treatment

All patients were treated with definitive-intent surgery and NCT. A total of 142 patients did not receive adjuvant chemotherapy. 5-FU-based chemotherapy was delivered concurrently with radiation in forms of three-dimensional conformal radiotherapy (3D-CRT), intensity-modulated radiotherapy (IMRT) or volumetric modulated arc therapy (VMAT). The clinical target volume (CTV) included the lymphatic drainage areas, i.e., mesorectal, internal iliac, and presacral lymph nodes. After one cycle of FU-based chemotherapy (5FU/xeloda or 5FU + oxaliplatin/xeloda + oxaliplatin), NCRT was administrated with other cycles 2–4 concurrent with radiotherapy. Afterwards, 500 patients received adjuvant chemotherapy.

### Statistical analysis

The primary endpoint for the present study was disease-free survival (DFS), defined as the duration from the first day of therapy to the date of confirmed relapse of disease. DFS was censored at the date of death from other causes, or the date of the last follow-up visit for disease-free patients. LRFS, and distant metastasis-free survival (DMFS), (definitions of LRFS, and DMFS provided in Supplementary Appendix) and ypStage were the secondary endpoints. Clinicopathological differences between the pMMR and dMMR groups stratified by neoadjuvant treatment groups were compared with the Mann–Whitney *U* test for continuous variables and *χ*^2^ test (or Fisher’s exact test, if appropriate) for categorical data. Correlation of MMR status with survival outcomes was analyzed with a propensity score matching technique to address potential heterogeneity in clinical characteristics between MMR-proficient (pMMR) and MMR-deficient (dMMR) groups (statistical analysis of the propensity score match in Supplementary method). Survival analyses were conducted separately for pMMR and dMMR groups. The survival curves were generated by Kaplan–Meier method and univariable survival between these subgroups was compared by using the log-rank test. Moreover, local regional recurrence (LR) and non-LR-associated death, and separately DM and non-DM-associated death were considered competing events (statistical analysis of the competing risk estimate is shown in Supplementary Appendix). Cox proportional-hazards regression analysis, which adjusted for clinicopathologic covariates (including age, gender, clinical T or N stage, localization, and NRT) was used to calculate *P* values, hazard ratios (HRs), and 95% confidence intervals (CIs). Two-sided *P* values of <0.05 were defined as statistical significance. All statistical analyses were performed using R software (version 3.5.1; http://www.Rproject.org).

### Reporting summary

Further information on research design is available in the Nature Research Reporting Summary linked to this article.

## Supplementary information

supplementary appendix

reporting summary

## Data Availability

The data for patients with dMMR tumors is included in the supplementary information files. Other datasets used and/or analyzed during the current study are available from the corresponding author on reasonable request.

## References

[1] Benson AB (2018). Rectal cancer, version 2.2018, NCCN Clinical Practice Guidelines in Oncology. J. Natl Compr. Cancer Netw..

[2] Lindor NM (2002). Immunohistochemistry versus microsatellite instability testing in phenotyping colorectal tumors. J. Clin. Oncol..

[3] Ribic CM (2003). Tumor microsatellite-instability status as a predictor of benefit from fluorouracil-based adjuvant chemotherapy for colon cancer. N. Engl. J. Med..

[4] Sargent DJ (2010). Defective mismatch repair as a predictive marker for lack of efficacy of fluorouracil-based adjuvant therapy in colon cancer. J. Clin. Oncol..

[5] Fotheringham S, Mozolowski GA, Murray EMA, Kerr DJ (2019). Challenges and solutions in patient treatment strategies for stage II colon cancer. Gastroenterol. Rep..

[6] Hasan, S. et al. Microsatellite instability (MSI) as an independent predictor of pathologic complete response (PCR) in locally advanced rectal cancer: a National Cancer Database (NCDB) analysis. *Ann. Surg.*10.1097/SLA.0000000000003051 (2018). 10.1097/SLA.0000000000003051 PMC7418064 30216221

[7] Li LS (2009). DNA mismatch repair (MMR)-dependent 5-fluorouracil cytotoxicity and the potential for new therapeutic targets. Br. J. Pharm..

[8] Samowitz WS (2009). Microsatellite instability and survival in rectal cancer. Cancer Causes Control.

[9] Vilar E, Gruber SB (2010). Microsatellite instability in colorectal cancer-the stable evidence. Nat. Rev. Clin. Oncol..

[10] Shin JS, Tut TG, Yang T, Lee CS (2013). Radiotherapy response in microsatellite instability related rectal cancer. Korean J. Pathol..

[11] Sauer R (2012). Preoperative versus postoperative chemoradiotherapy for locally advanced rectal cancer: results of the German CAO/ARO/AIO-94 randomized phase III trial after a median follow-up of 11 years. J. Clin. Oncol..

[12] van Gijn W (2011). Preoperative radiotherapy combined with total mesorectal excision for resectable rectal cancer: 12-year follow-up of the multicentre, randomised controlled TME trial. Lancet Oncol..

[13] Bruheim K (2010). Late side effects and quality of life after radiotherapy for rectal cancer. Int. J. Radiat. Oncol. Biol. Phys..

[14] Bosse D (2016). PROSPECT eligibility and clinical outcomes: results from the Pan-Canadian Rectal Cancer Consortium. Clin. Colorectal Cancer.

[15] Deng Y (2016). Modified FOLFOX6 with or without radiation versus fluorouracil and leucovorin with radiation in neoadjuvant treatment of locally advanced rectal cancer: initial results of the Chinese FOWARC Multicenter, Open-Label, Randomized Three-Arm Phase III Trial. J. Clin. Oncol..

[16] Le DT (2015). PD-1 blockade in tumors with mismatch-repair deficiency. N. Engl. J. Med..

[17] Deng, Y. et al. Neoadjuvant modified FOLFOX6 with or without radiation versus fluorouracil plus radiation for locally advanced rectal cancer: final results of the Chinese FOWARC trial. *J. Clin. Oncol.* JCO1802309. 10.1200/JCO.18.02309 (2019). 10.1200/JCO.18.02309 PMC6881102 31557064

[18] Zhang, J. et al. Neoadjuvant chemotherapy with mFOLFOXIRI without routine use of radiotherapy for locally advanced rectal cancer. *Clin. Colorectal Cancer*. 10.1016/j.clcc.2019.07.001 (2019). 10.1016/j.clcc.2019.07.001 31378655

[19] de Rosa N (2016). DNA mismatch repair deficiency in rectal cancer: benchmarking its impact on prognosis, neoadjuvant response prediction, and clinical cancer genetics. J. Clin. Oncol..

[20] Du C, Zhao J, Xue W, Dou F, Gu J (2013). Prognostic value of microsatellite instability in sporadic locally advanced rectal cancer following neoadjuvant radiotherapy. Histopathology.

[21] Huh JW (2016). Mismatch repair gene expression as a predictor of tumor responses in patients with rectal cancer treated with preoperative chemoradiation. Medicine.

[22] Meillan, N. et al. Mismatch repair system deficiency is associated with response to neoadjuvant chemoradiation in locally advanced rectal cancer. *Int. J. Radiat. Oncol. Biol. Phys*. 10.1016/j.ijrobp.2019.07.057 (2019). 10.1016/j.ijrobp.2019.07.057 31404579

[23] Cercek, A. et al. Mismatch repair-deficient rectal cancer and resistance to neoadjuvant chemotherapy. *Clin. Cancer Res.*10.1158/1078-0432.CCR-19-3728 (2020). 10.1158/1078-0432.CCR-19-3728 PMC7348681 32144135

[24] Ferrari L, Fichera A (2015). Neoadjuvant chemoradiation therapy and pathological complete response in rectal cancer. Gastroenterol. Rep..

[25] Bertagnolli MM (2009). Microsatellite instability predicts improved response to adjuvant therapy with irinotecan, fluorouracil, and leucovorin in stage III colon cancer: Cancer and Leukemia Group B Protocol 89803. J. Clin. Oncol..

[26] Zaanan A (2010). Impact of p53 expression and microsatellite instability on stage III colon cancer disease-free survival in patients treated by 5-fluorouracil and leucovorin with or without oxaliplatin. Ann. Oncol..

[27] Guinney J (2015). The consensus molecular subtypes of colorectal cancer. Nat. Med..

[28] Jiang D (2019). Mutation in BRAF and SMAD4 associated with resistance to neoadjuvant chemoradiation therapy in locally advanced rectal cancer. Virchows Arch..

[29] Zhang J, Cai J, Deng Y, Wang H (2019). Complete response in patients with locally advanced rectal cancer after neoadjuvant treatment with nivolumab. Oncoimmunology.

[30] Karagkounis, G. et al. Prognostic implications of pathological response to neoadjuvant chemoradiation in pathologic stage III rectal cancer. *Ann. Surg.*10.1097/SLA.0000000000002719 (2018). 10.1097/SLA.0000000000002719 31082910

[31] Sinukumar S, Patil P, Engineer R, Desouza A, Saklani A (2014). Clinical outcome of patients with complete pathological response to neoadjuvant chemoradiotherapy for locally advanced rectal cancers: the Indian scenario. Gastroenterol. Res. Pract..

[32] Delitto D (2018). Prognostic value of clinical vs. pathologic stage in rectal cancer patients receiving neoadjuvant therapy. J. Natl Cancer Inst..

[33] Glynne-Jones R (2018). Rectal cancer: ESMO Clinical Practice Guidelines for diagnosis, treatment and follow-up. Ann. Oncol..

[34] Ludmir EB, Palta M, Willett CG, Czito BG (2017). Total neoadjuvant therapy for rectal cancer: an emerging option. Cancer.

[35] Cercek A (2018). Adoption of total neoadjuvant therapy for locally advanced rectal cancer. JAMA Oncol..

[36] Austin PC (2009). The relative ability of different propensity score methods to balance measured covariates between treated and untreated subjects in observational studies. Med. Decis. Mak..

[37] Oh CR (2018). Prognostic value of the microsatellite instability status in patients with stage II/III rectal cancer following upfront surgery. Clin. Colorectal Cancer.

[38] Stadler ZK (2016). Reliable detection of mismatch repair deficiency in colorectal cancers using mutational load in next-generation sequencing panels. J. Clin. Oncol..

[39] Altman DG, McShane LM, Sauerbrei W, Taube SE (2012). Reporting Recommendations for Tumor Marker Prognostic Studies (REMARK): explanation and elaboration. PLoS Med..

