# Supplementary material for: Association of mismatch repair status with survival and response to neoadjuvant chemo(radio)therapy in rectal cancer
Source: NPJ Precis Oncol. 2020 Sep 7;4:26. doi: 10.1038/s41698-020-00132-5 (PMC7477257; doi:10.1038/s41698-020-00132-5)
Supplement: Supplementary file 1 — supplementary appendix [file 41698_2020_132_MOESM1_ESM.docx]

**Association of Mismatch Repair Status With Survival and Response to Neoadjuvant Chemo(radio)therapy in Rectal Cancer**

**INDEX OF SUPPLEMENTARY INFORMATION**

**SUPPLEMENTARY METHODS1**

**SUPPLEMENTARY TABLES 2**

**SUPPLEMENTARY FIGURES 11**

**SUPPLEMENTARY CASE REPORT 18**

**Supplementary methods**

**Definitions of local recurrence-free survival (LRFS), and distant metastasis-free survival (DMFS).**

LRFS was defined as the first day of therapy to the date first local relapse within pelvic; DMFS was defined as the first day of therapy to the date first distant relapse.

**Competing risk estimate**

Cumulative incidence function (CIF) was used to show the probability of each event and the differences between the groups were estimated using Gray’s test.

**Statistical analysis of the propensity score match**

Correlation of mismatch repair (MMR) status with survival outcomes was analyzed with a propensity score matching technique to address potential heterogeneity in clinical characteristics between MMR-proficient (pMMR) and MMR-deficient (dMMR) groups. The propensity model included potential prognostic parameters including sex, clinical T/N stage, tumor location and age that was significantly distributed unequally among the two groups on the multivariate logistic regression model. The propensity scores matching approach based on the caliper method was conducted with a ratio of 2:1 (2 pMMR patients matched to 1 dMMR patient) to select one sample with well-balanced baseline characteristics. Cases with missing data were dropped from the propensity score model, and only parameters with <5% missing data were included.

Supplementary Table 1. Detailed clinicopathologic information of patients with dMMR tumors

| Case | Gender | Age  (y) | Distance  (cm) | NCT regimen | NRT | ACT | cT | cN | cStage | pCR | pT | pN | pStage | Grade | Relapse | Death |
| --- | --- | --- | --- | --- | --- | --- | --- | --- | --- | --- | --- | --- | --- | --- | --- | --- |
| 1 | male | 52 | 4 | 2 | Yes | Yes | T3 | N1 | III | No | T3 | N0 | II | 1 | Yes | No |
| 2 | female | 61 | 3 | 2 | Yes | Yes | T3 | N1 | III | No | T3 | N2 | III | 2 | Yes | Yes |
| 3 | female | 44 | 2 | 2 | Yes | Yes | T3 | N2 | III | No | T3 | N2 | III | 2 | Yes | No |
| 4 | male | 42 | 5 | 2 | Yes | Yes | T3 | N0 | II | No | T3 | N0 | II | 3 | Yes | No |
| 5 | male | 38 | 4 | 1 | Yes | Yes | T3 | N0 | II | No | T3 | N0 | II | 2 | Yes | No |
| 6 | female | 46 | 10 | 2 | No | Yes | T2 | N1 | III | Yes | T0 | N0 | 0 | 1 | No | No |
| 7 | female | 42 | 2.5 | 2 | Yes | No | T3 | N0 | II | No | T2 | N0 | I | 2 | No | No |
| 8 | male | 32 | 1.7 | 2 | Yes | No | T3 | N0 | II | Yes | T0 | N0 | 0 | 2 | No | No |
| 9 | female | 59 | 4 | 1 | Yes | Yes | T4 | N0 | II | Yes | T0 | N0 | 0 | 1 | Yes | Yes |
| 10 | male | 40 | 4 | 2 | Yes | Yes | T3 | N0 | II | No | T3 | N0 | II | 2 | Yes | No |
| 11 | male | 30 | 13.8 | 1 | Yes | Yes | T4 | N0 | II | No | T3 | N0 | II | 2 | No | No |
| 12 | female | 73 | 4.9 | 2 | No | Yes | T3 | N0 | II | No | T2 | N1 | III | 1 | No | No |
| 13 | male | 22 | 8 | 2 | No | Yes | T3 | N0 | II | No | T1 | N0 | I | 1 | No | No |
| 14 | male | 56 | 1.8 | 2 | Yes | Yes | T3 | N0 | II | No | T3 | N2 | III | 3 | No | No |
| 15 | male | 48 | 2 | 2 | Yes | Yes | T3 | N2 | III | No | T3 | N1 | III | 2 | Yes | No |
| 16 | male | 57 | 5 | 2 | Yes | Yes | T3 | N0 | II | No | T3 | N0 | II | NS | No | No |
| 17 | male | 37 | 1 | 2 | Yes | Yes | 4 | N0 | II | No | T3 | N0 | II | 2 | Yes | Yes |
| 18 | male | 55 | 2 | 2 | Yes | Yes | T3 | N0 | II | No | T3 | N0 | II | 2 | No | No |
| 19 | male | 58 | 3.5 | 2 | Yes | Yes | T3 | N0 | II | No | T3 | N2 | III | 2 | Yes | Yes |
| 20 | male | 56 | 3 | 1 | Yes | No | T4 | N1 | III | No | T3 | N0 | II | 2 | No | No |
| 21 | female | 64 | 3.1 | 2 | No | No | T3 | N1 | III | No | T2 | N0 | I | 2 | No | Yes |
| 22 | male | 53 | 5 | 2 | No | No | T3 | N1 | III | No | T3 | N1 | III | 2 | No | No |
| 23 | female | 47 | 5.2 | 2 | No | No | T3 | N1 | III | No | T3 | N0 | II | 2 | No | No |
| 24 | male | 28 | 7.1 | 2 | Yes | No | T3 | N2 | III | No | T3 | N0 | II | 1 | Yes | No |
| 25 | male | 55 | 8.6 | 2 | Yes | Yes | T4 | N2 | III | No | T3 | N0 | II | 2 | Yes | Yes |
| 26 | male | 33 | 7.5 | 1 | Yes | Yes | T4 | N2 | III | No | T3 | N2 | III | 3 | Yes | Yes |
| 27 | female | 69 | 2 | 1 | Yes | Yes | T4 | N1 | III | No | T3 | N0 | II | 1 | Yes | No |
| 28 | female | 66 | 11 | 1 | Yes | Yes | T4 | N1 | III | No | T3 | N1 | III | 2 | Yes | Yes |
| 29 | male | 59 | 4 | 1 | Yes | Yes | T3 | N2 | III | No | T3 | N0 | II | 1 | Yes | No |
| 30 | female | 42 | 2.9 | 2 | No | Yes | T4 | N2 | III | No | T4 | N1 | III | 2 | Yes | No |
| 31 | male | 29 | 5 | 3 | No | Yes | T4 | N1 | III | No | T3 | N1 | III | 1 | No | No |
| 32 | male | 57 | 4.5 | 2 | No | Yes | T4 | N1 | III | No | T3 | N0 | II | 3 | No | No |
| 33 | female | 88 | 10 | 2 | No | Yes | T3 | N1 | III | No | T3 | N0 | II | 2 | No | No |
| 34 | male | 59 | 3.1 | 2 | No | Yes | T3 | N1 | III | No | T2 | N1 | III | 1 | No | No |
| 35 | male | 40 | 10 | 2 | No | Yes | T3 | N1 | III | No | T2 | N0 | I | 1 | No | No |
| 36 | male | 67 | 4 | 1 | Yes | Yes | T3 | N0 | II | No | T2 | N0 | I | 2 | No | No |
| 37 | male | 59 | 3.1 | 2 | Yes | Yes | T3 | N1 | III | No | T3 | N0 | II | 3 | No | No |
| 38 | male | 51 | 7.2 | 3 | No | Yes | T4 | N2 | III | No | T4 | N0 | II | 1 | No | No |
| 39 | male | 39 | 6.8 | 2 | Yes | Yes | T3 | N2 | III | No | T2 | N0 | I | 1 | No | No |
| 40 | male | 31 | 8 | 2 | Yes | Yes | T4 | N2 | III | No | T3 | N1 | III | 2 | No | No |
| 41 | male | 42 | 8 | 1 | No | Yes | T4 | N1 | III | No | T3 | N1 | III | 2 | No | No |
| 42 | female | 48 | 11 | 2 | Yes | Yes | T4 | N1 | III | Yes | T0 | N0 | 0 | 3 | No | No |
| 43 | female | 40 | 11 | 3 | No | Yes | T3 | N2 | III | No | T3 | N0 | II | 3 | No | No |
| 44 | male | 54 | 2.8 | 3 | No | Yes | T3 | N1 | III | No | T2 | N0 | I | 2 | No | No |
| 45 | male | 47 | 8 | 2 | Yes | Yes | T3 | N1 | III | No | T3 | N0 | II | 2 | No | No |
| 46 | male | 53 | 4.9 | 2 | No | Yes | T3 | N2 | III | No | T3 | N0 | II | 1 | No | No |
| 47 | male | 66 | 5.2 | 2 | Yes | Yes | T3 | N2 | III | Yes | T0 | N0 | 0 | 1 | No | No |
| 48 | male | 66 | 10.5 | 1 | Yes | Yes | T4 | N2 | III | No | T3 | N1 | III | 2 | No | No |
| 49 | male | 67 | 3.4 | 2 | Yes | Yes | T3 | N1 | III | No | T3 | N0 | II | 2 | No | No |
| 50 | male | 56 | 2.5 | 3 | No | Yes | T4 | N1 | III | Yes | T0 | N0 | 0 | 2 | No | No |
| 51 | male | 42 | 4.1 | 2 | Yes | Yes | T3 | N2 | III | No | T3 | N0 | II | 2 | No | No |
| 52 | male | 31 | 10 | 2 | No | Yes | T3 | N2 | III | No | T3 | N1 | III | 3 | No | No |
| 53 | male | 41 | 7 | 2 | Yes | Yes | T3 | N1 | III | No | T4 | N2 | III | 2 | No | No |
| 54 | female | 44 | 2 | 2 | Yes | Yes | T3 | N1 | III | No | T2 | N1 | III | 2 | No | No |
| 55 | male | 57 | 7 | 2 | Yes | Yes | T3 | N2 | III | No | T4 | N0 | II | 3 | No | No |
| 56 | male | 56 | 9 | 2 | Yes | Yes | T3 | N2 | III | No | T2 | N0 | I | NS | No | No |
| 57 | male | 46 | 8 | 2 | Yes | Yes | T3 | N1 | III | No | T3 | N1 | III | 2 | No | No |
| 58 | female | 73 | 1.5 | 2 | Yes | Yes | T4 | N1 | III | No | T4 | N0 | II | 2 | No | No |
| 59 | female | 36 | 1 | 2 | Yes | Yes | T3 | N1 | III | No | T3 | N2 | III | 2 | No | No |
| 60 | male | 45 | 5 | 2 | Yes | Yes | T3 | N1 | III | No | T4 | N2 | III | 3 | Yes | Yes |
| 61 | male | 45 | 5 | 1 | Yes | Yes | T4 | N1 | III | No | T4 | N0 | II | 2 | Yes | Yes |
| 62 | female | 33 | 3.8 | 2 | Yes | Yes | T3 | N2 | III | No | T1 | N0 | I | 1 | No | No |
| 63 | male | 46 | 6.5 | 2 | No | Yes | T3 | N1 | III | No | T3 | N0 | II | 2 | No | No |
| 64 | male | 41 | 7 | 2 | No | Yes | T4 | N2 | III | No | T3 | N0 | II | 2 | No | No |
| 65 | female | 34 | 7 | 2 | Yes | Yes | T3 | N1 | III | No | T4 | N0 | II | 3 | Yes | Yes |
| 66 | male | 67 | 8 | 2 | Yes | No | T4 | N0 | II | No | T2 | N0 | I | 1 | No | No |

dMMR, deficient mismatch repair; NCT regimen, neoadjuvant chemotherapy regimen (1= fluorouracil/xeloda, 2= fluorouracil/xeloda+ oxaliplatin, 3= fluorouracil+ oxaliplatin); NRT, neoadjuvant radiotherapy; ACT, adjuvant chemotherapy; cT, clinical T classification; cN, clinical N classification; cStage, clinical stage; pCR, pathologic complete response; pT, pathologic T classification; pN, pathologic N classification; pStage, pathologic stage; Grade, histology grade (1=well differentiated; 2=moderate differentiated; 3=low differentiated, NS=not sure).

Supplementary Table 2 Baseline Characteristics of patients with stage III disease and pMMR tumors

| Characteristics | No NRT, n=301 | NRT, n=439 | P value |
| --- | --- | --- | --- |
| Sex (%) |  |  | 0.064 |
| Male | 218 (72.4) | 289 (65.8) |  |
| Female | 83 (27.6) | 150 (34.2) |  |
| Age (y) (%) |  |  | 0.708 |
| Mean (SD) | 56 (± 13) | 57 (± 11) |  |
| ≤60 | 187 (62.1) | 269 (61.3) |  |
| >60 | 114 (37.9) | 170 (38.7) |  |
| Comorbidity (%) |  |  | 0.725 |
| Yes | 74 (24.6) | 102 (23.2) |  |
| No | 227 (75.4) | 337 (76.8) |  |
| BMI |  |  | 0.632 |
| Mean (SD) | 22.6 (3.6) | 22.7 (3.0) |  |
| Clinical T stage (%) |  |  | 0.007^*^ |
| T1 | 1 (0.3) | 0 (0) |  |
| T2 | 6 (2.0) | 9 (2.1) |  |
| T3 | 240 (79.7) | 309 (70.4) |  |
| T4 | 54 (17.9) | 121 (27.6) |  |
| Clinical N stage (%) |  |  | 0.140 |
| N1 | 182 (60.5) | 289 (65.8) |  |
| N2 | 119 (39.5) | 150 (34.2) |  |
| Distance from anal verge (%) |  |  | <0.001^*^ |
| ≤5 cm | 115 (38.2) | 206 (46.9) |  |
| >5 and ≤10 cm | 152 (50.5) | 220 (50.1) |  |
| >10 cm | 34 (11.3) | 13 (3.0) |  |
| ypStage (%) |  |  | 0.003^*^ |
| 0-I | 82 (27.2) | 165 (37.6) |  |
| II-III | 219 (72.8) | 274 (62.4) |  |
| pCR | 9 (3.0) | 40 (9.1) | 0.001^*^ |
| Differentiation (%) |  |  | 0.006^*^ |
| Well | 67 (22.3) | 61 (13.9) |  |
| Moderately | 200 (66.4) | 306 (69.7) |  |
| Poorly | 30 (10.0) | 55 (12.5) |  |
| NS | 4 (1.3) | 17 (3.9) |  |
| Adjuvant chemotherapy (ACT) (%) |  |  | 0.046^*^ |
| Yes | 271 (90.0) | 373 (85.0) |  |
| No | 30 (10.0) | 66 (15.0) |  |

^*,^ statistically significant; pMMR, proficient mismatch repair; SD, standard deviation; BMI, body mass index; NRT, neoadjuvant radiotherapy; pCR, pathologic complete response; NS, not sure.

Supplementary Table 3 LRFS and DMFS by MMR status in univariable and multivariable analysis adjusted for clinical characteristics

| Survival | 5-Year rates (%) | Univariable |  | Multivariable | | |
| --- | --- | --- | --- | --- | --- | --- |
|  |  | P value |  | HR | 95%CI | P value |
| LRFS |  |  |  |  |  |  |
| dMMR |  |  |  |  |  |  |
| NCRT | 91 | 0.2 |  | / | / | 1.00 |
| NCT | 100 |  |  |  |  |  |
| pMMR |  |  |  |  |  |  |
| NCRT | 91 | 0.002^*^ |  | 0.403 | 0.241-0.673 | 0.001^*^ |
| NCT | 84 |  |  |  |  |  |
| DMFS |  |  |  |  |  |  |
| dMMR |  |  |  |  |  |  |
| NCRT | 44 | 0.03^*^ |  | 8.828 | 1.081-72.064 | 0.042^*^ |
| NCT | 92 |  |  |  |  |  |
| pMMR |  |  |  |  |  |  |
| NCRT | 72 | 0.1 |  | 0.818 | 0.613-1.090 | 0.169 |
| NCT | 67 |  |  |  |  |  |

^*,^ statistically significant; dMMR, deficient mismatch repair; pMMR, proficient mismatch repair; LRFS, local recurrence-free survival; DMFS, distant metastasis-free survival; NRT, neoadjuvant radiotherapy; NCRT, neoadjuvant chemoradiotherapy; NCT, neoadjuvant chemotherapy; HR, hazard ratios; CI, confidential interval.

Supplementary Table 4 Survival by neoadjuvant radiotherapy for pMMR patients in univariate and multivariate analysis adjusted for clinical characteristics

| Univariate | | | | | | | | | | | | | |
| --- | --- | --- | --- | --- | --- | --- | --- | --- | --- | --- | --- | --- | --- |
|  | DFS | | | | LRFS | | | | DMFS | | | | |
|  | Hazard Ratio  (95% CI) | | P value | | Hazard Ratio  (95% CI) | | P value | | Hazard Ratio  (95% CI) | | P value | | |
| NRT |  | |  | |  | |  | |  | |  | | |
| With vs. without | 0.762 (0.594-0.978) | | 0.033^*^ | | 0.479 (0.296- 0.774) | | 0.002^*^ | | 0.794 (0.603-1.045) | | 0.100 | | |
| Multivariate | | | | | | | | | | | | | |
| NRT | |  | |  | |  | |  | |  | | |  |
| with vs. without | | 0.763 (0.587-0.991) | | 0.043^*^ | | 0.403 (0.241-0.673) | | 0.001^*^ | | 0.818 (0.613-1.090) | | | 0.169 |
| Age | |  | |  | |  | |  | |  | |  | |
| >60y vs. ≤60y | | 0.954 (0.738-1.234) | | 0.720 | | 0.910 (0.549-1.507) | | 0.714 | | 0.895 (0.674-1.190) | | 0.446 | |
| Sex | |  | |  | |  | |  | |  | |  | |
| Female vs. male | | 0.872 (0.662-1.147) | | 0.327 | | 0.619 (0.352-1.088) | | 0.095 | | 0.996 (0.740-1.338) | | 0.973 | |
| Clinical T stage | |  | |  | |  | |  | |  | |  | |
| T1-3 vs. T4 | | 1.254 (0.933-1.686) | | 0.134 | | 2.348 (1.383-3.986) | | 0.002^*^ | | 1.085 (0.776-1.517) | | 0.631 | |
| Clinical N stage | |  | |  | |  | |  | |  | |  | |
| N0 | | 1.000 | |  | | 1.000 | |  | | 1.000 | |  | |
| N1 | | 0.995 (0.715-1.385) | | 0.976 | | 0.970 (0.496-1.896) | | 0.929 | | 0.988 (0.684-1.427) | | 0.950 | |
| N2 | | 1.451 (1.018-2.070) | | 0.040^*^ | | 2.111 (1.073-4.161) | | 0.030^*^ | | 1.445 (0.977-2.139) | | 0.065 | |
| Localization | |  | |  | |  | |  | |  | |  | |
| Low | | 1.000 | |  | | 1.000 | |  | | 1.000 | |  | |
| Middle | | 0.864 (0.644-1.126) | | 0.280 | | 0.398 (0.230-0.690) | | 0.001^*^ | | 1.015 (0.760-1.356) | | 0.917 | |
| High | | 1.402 (0.851-2.310) | | 0.185 | | 0.843 (0.372-1.911) | | 0.683 | | 1.409 (0.796-2.493) | | 0.239 | |

^*,^ statistically significant; pMMR, proficient mismatch repair; DFS. disease-free survial; LRFS, local recurrence-free survival; DMFS, distant metastasis-free survival; NRT, neoadjuvant radiotherapy; CI, confidential interval.

Supplementary Table 5 Baseline Characteristics for the remaining cohort

| Characteristics | pMMR (n=889) | | | |  | dMMR (n=60) | | |
| --- | --- | --- | --- | --- | --- | --- | --- | --- |
|  | No NCRT  (n=389) | | NCT  (n=500) | p |  | No NCRT  (n=19) | NCT  (n=41) | p |
| Age (y, %) |  | |  | 0.418 |  |  |  | 0.769 |
| Mean (SD) | 56 (13) | | 57 (11) |  |  | 47 (15) | 47 (12) |  |
| ≤60 | 243 (62.5) | | 296 (59.2) |  |  | 16 (84.2) | 33 (80.5) |  |
| >60 | 155 (37.5) | | 204 (40.8) |  |  | 3 (15.8) | 8 (19.5) |  |
| Sex (%) |  | |  | 0.382 |  |  |  | 0.754 |
| Male | 274 (70.4) | | 338 (67.6) |  |  | 13 (68.4) | 31 (75.6) |  |
| Female | 115 (29.6) | | 160 (32.4) |  |  | 6 (31.6) | 10 (24.4) |  |
| BMI (%) |  | |  | 0.468 |  |  |  | 0.713 |
| Mean (SD) | 22.4 (3.5) | | 22.6 (3.0) |  |  | 22.2 (2.9) | 22.3 (3.1) |  |
| Comorbidity (%) |  | |  | 0.910 |  |  |  | 1.000 |
| Yes | 89 (22.9) | | 116 (23.2) |  |  | 3 (15.8) | 8 (19.5) |  |
| No | 300 (77.1) | | 384 (76.8) |  |  | 16 (84.2) | 33 (80.5) |  |
| Clinical T stage (%) |  | |  | 0.010^*^ |  |  |  | 0.856 |
| T1 | 1 (0.3) | | 0 (0) |  |  | 0 (0) | 0 (0) |  |
| T2 | 6 (1.5) | | 8 (1.6) |  |  | 0 (0) | 0 (0) |  |
| T3 | 312 (80.2) | | 360 (72.0) |  |  | 13 (68.4) | 29 (70.7) |  |
| T4 | 70 (18.0) | | 132 (26.4) |  |  | 6 (31.6) | 12 (29.3) |  |
| Clinical N stage (%) |  | |  | 0.145 |  |  |  | 0.254 |
| N0 | 95 (24.4) | | 101 (20.2) |  |  | 2 (10.5) | 12 (29.3) |  |
| N1 | 177 (45.5) | | 259 (51.8) |  |  | 11 (57.9) | 16 (39.0) |  |
| N2 | 117 (30.1) | | 140 (28.0) |  |  | 6 (31.6) | 13 (31.7) |  |
| Distance from anal verge (%) | |  |  | <0.001^*^ |  |  |  | 0.375 |
| ≤5 cm | 156 (40.1) | | 256 (51.2) |  |  | 9 (47.4) | 26 (63.4) |  |
| >5 and ≤10 cm | 193 (49.6) | | 231 (46.2) |  |  | 9 (47.4) | 12 (29.3) |  |
| >10 cm | 40 (10.3) | | 13 (2.6) |  |  | 1 (5.2) | 3 (7.3) |  |
| ypStage (%) |  | |  | 0.172 |  |  |  | 0.711 |
| 0-I | 105 (27.0) | | 156 (31.2) |  |  | 4 (21.1) | 6 (14.6) |  |
| II-III | 284 (73.0) | | 344 (68.8) |  |  | 15 (78.9) | 35 (85.4) |  |
| Differentiation (%) |  | |  | <0.001^*^ |  |  |  | 0.377 |
| Well | 89 (22.9) | | 63 (12.6) |  |  | 7 (36.8) | 7 (17.1) |  |
| Moderately | 256 (65.8) | | 366 (73.2) |  |  | 9 (47.4) | 25 (61.0) |  |
| Poorly | 39 (10.0) | | 55 (11.0) |  |  | 3 (15.8) | 7 (17.1) |  |
| NS | 5 (1.3) | | 16 (3.2) |  |  | 0 (0) | 2 (4.9) |  |
| Adjuvant chemotherapy (%) |  | |  | 0.137 |  |  |  | 0.668 |
| Yes | 344 (88.4) | | 425 (85.0) |  |  | 16 (84.2) | 37 (90.2) |  |
| No | 45 (11.6) | | 75 (15.0) |  |  | 3 (15.8) | 4 (9.8) |  |

^*,^ statistically significant; dMMR, deficient mismatch repair; pMMR, proficient mismatch repair; SD, standard deviation; BMI, body mass index; NRT, neoadjuvant radiotherapy; NCRT, neoadjuvant chemoradiotherapy; NCT, neoadjuvant chemotherapy; NS, not sure.

Supplementary Table 6 Survival by neoadjuvant radiotherapy for remaining cohort with dMMR tumors in multivariate analysis adjusted for clinical characteristics

| Multivariate | | | | | | | |
| --- | --- | --- | --- | --- | --- | --- | --- |
|  | DFS | | | | DMFS | | |
|  | Hazard Ratio  (95% CI) | | P value | | Hazard Ratio  (95% CI) | | P value |
| NRT | |  | |  | |  |  |
| with vs. without | | 11.113 (1.395-88.520) | | 0.023^*^ | | 9.296 (1.123-76.934) | 0.039^*^ |
| Age | |  | |  | |  |  |
| >60y vs. ≤60y | | 0.756 (0.162-3.524) | | 0.722 | | 0.759 (0.147-3.912) | 0.742 |
| Sex | |  | |  | |  |  |
| Female vs. male | | 1.436 (0.466-4.422) | | 0.528 | | 1.692 (0.520-5.504) | 0.382 |
| Clinical T stage | |  | |  | |  |  |
| T2-3 vs T4 | | 3.225 (1.008-10.316) | | 0.048^*^ | | 3.439 (0.927-12.753) | 0.065 |
| Clinical N stage | |  | |  | |  |  |
| N0 | | 1.000 | |  | | 1.000 |  |
| N1 | | 0.760 (0.209-2.769) | | 0.678 | | 1.041 (0.255-4.241) | 0.956 |
| N2 | | 1.940 (0.499-7.538) | | 0.339 | | 2.469 (0.550-11.084) | 0.238 |
| Localization | |  | |  | |  |  |
| Low | | 1.000 | |  | | 1.000 |  |
| Middle | | 0.366 (0.089-1.506) | | 0.163 | | 0.194 (0.033-1.134) | 0.069 |
| High | | 0.242 (0.025-2.392) | | 0.225 | | 0.278 (0.027-2.845) | 0.281 |

Among 59 patients with dMMR tumors, there were only 4 local recurrence events; thus, data of local recurrence-free survival were not shown in Supplementary Table 6.

^*,^ statistically significant; dMMR, deficient mismatch repair; DFS, disease-free survival; DMFS, distant metastasis-free survival; NRT, neoadjuvant radiotherapy; CI, confidential interval.

Supplementary Table 7 Survival by neoadjuvant radiotherapy for remaining cohort with pMMR tumors in univariate and multivariate analysis adjusted for clinical characteristics

| Univariate | | | | | | | | | | | | | |
| --- | --- | --- | --- | --- | --- | --- | --- | --- | --- | --- | --- | --- | --- |
|  | DFS | | | | LRFS | | | | DMFS | | | | |
|  | Hazard Ratio  (95% CI) | | P value | | Hazard Ratio  (95% CI) | | P value | | Hazard Ratio  (95% CI) | | P value | | |
| NRT |  | |  | |  | |  | |  | |  | | |
| With vs. without | 0.767 (0.594-0.989) | | 0.041 | | 0.497 (0.303-0.815) | | 0.006^*^ | | 0.788 (0.596-1.041) | | 0.094 | | |
| Multivariate | | | | | | | | | | | | | |
| Neoadjuvant RT | |  | |  | |  | |  | |  | | |  |
| with vs. without | | 0.709 (0.538-0.933) | | 0.0515 | | 0.438 (0.257-0.741) | | 0.002^*^ | | 0.801 (0.598-1.072) | | | 0.133 |
| Age | |  | |  | |  | |  | |  | |  | |
| >60y vs. ≤60y | | 0.968 (0.745-1.258) | | 0.809 | | 0.924 (0.551-1.552) | | 0.766 | | 0.901 (0.675-1.203) | | 0.480 | |
| Sex | |  | |  | |  | |  | |  | |  | |
| Female vs. male | | 0.909 (0.688-1.201) | | 0.501 | | 0.624 (0.348-1.117) | | 0.113 | | 1.042 (0.773-1.405) | | 0.786 | |
| Clinical T stage | |  | |  | |  | |  | |  | |  | |
| T1-3 vs. T4 | | 1.315 (0.973-1.777) | | 0.075 | | 2.296 (1.329-3.968) | | 0.003^*^ | | 1.171 (0.837-1.640) | | 0.357 | |
| Clinical N stage | |  | |  | |  | |  | |  | |  | |
| N0 | | 1.000 | |  | | 1.000 | |  | | 1.000 | |  | |
| N1 | | 1.056 (0.748-1.493) | | 0.755 | | 1.015 (0.498-2.068) | | 0.968 | | 1.044 (0.713-1.529) | | 0.824 | |
| N2 | | 1.461 (1.001-2.112) | | 0.044^*^ | | 2.072 (1.009-4.255) | | 0.047^*^ | | 1.465 (0.979-2.193) | | 0.064 | |
| Localization | |  | |  | |  | |  | |  | |  | |
| Low | | 1.000 | |  | | 1.000 | |  | | 1.000 | |  | |
| Middle | | 0.895 (0.683-1.172) | | 0.421 | | 0.448 (0.256-0.785) | | 0.005^*^ | | 1.011 (0.754-1.358) | | 0.938 | |
| High | | 1.413 (0.856-2.333) | | 0.176 | | 0.941 (0.412-2.156) | | 0.886 | | 1.342 (0.758-2.376) | | 0.312 | |

^*,^ statistically significant; pMMR, proficient mismatch repair; DFS, disease-free survival; LRFS, local recurrence-free survival; DMFS, distant metastasis-free survival; NRT, neoadjuvant radiotherapy; CI, confidential interval.

Supplementary Table 8 Survival by neoadjuvant radiotherapy for remaining cohort with stage III disease and pMMR tumors in univariable and multivariable analysis adjusted for clinical characteristics

| Univariate | | | | | | | | | | | |
| --- | --- | --- | --- | --- | --- | --- | --- | --- | --- | --- | --- |
|  | DFS | | | | LRFS | | | | | DMFS | |
|  | Hazard Ratio  (95% CI) | | P value | | Hazard Ratio  (95% CI) | | P value | | | Hazard Ratio  (95% CI) | P value |
| NRT |  | |  | |  | |  | | |  |  |
| With vs. without | 0.725 (0.546-0.964) | | 0.027^*^ | | 0.451 (0.262-0.778) | | 0.003^*^ | | | 0.802 (0.587-1.097) | 0.200 |
| Multivariate | | | | | | | | | | | |
| NRT | |  | |  | |  | |  |  | |  |
| with vs. without | | 0.731 (0.543-0.985) | | 0.039^*^ | | 0.423 (0.237-0.754) | | 0.004^*^ | 0.808 (0.583-1.119) | | 0.196 |
| Age | |  | |  | |  | |  |  | |  |
| >60y vs. ≤60y | | 0.994 (0.741-1.333) | | 0.967 | | 1.111 (0.633-1.950) | | 0.714 | 0.895 (0.643-1.233) | | 0.485 |
| Sex | |  | |  | |  | |  |  | |  |
| Female vs. male | | 1.050 (0.774-1.424) | | 0.756 | | 0.609 (0.318-1.167) | | 0.135 | 1.240 (0.895-1.716) | | 0.196 |
| Clinical T stage | |  | |  | |  | |  |  | |  |
| T2-3 vs T4 | | 1.267 (0.907-1.768) | | 0.165 | | 2.029 (1.107-3.718) | | 0.022^*^ | 1.165 (0.807-1.684) | | 0.415 |
| Clinical N stage | |  | |  | |  | |  |  | |  |
| N1 vs N2 | | 1.375 (1.027-1.841) | | 0.033^*^ | | 2.080 (1.187-3.643) | | 0.010^*^ | 1.402 (1.019-1.928) | | 0.038^*^ |
| Localization | |  | |  | |  | |  |  | |  |
| Low | | 1.000 | |  | | 1.000 | |  | 1.000 | |  |
| Middle | | 0.912 (0.675-1.232) | | 0.548 | | 0.474 (0.258-0.870) | | 0.016^*^ | 1.027 (0.740-1.427) | | 0.872 |
| High | | 1.434 (0.835-2.465) | | 0.192 | | 1.036 (0.427-2.514) | | 0.937 | 1.270 (0.677-2.382) | | 0.456 |

^*,^ statistically significant; pMMR, proficient mismatch repair; DFS, disease-free survival; LRFS, local recurrence-free survival; DMFS, distant metastasis-free survival; NRT, neoadjuvant radiotherapy; CI, confidential interval.

**Supplementary Figure 1:** Survival Kaplan-Meier curves after propensity score matching (a) Disease-free survival (DFS), (b) Local recurrence-free survival (LRFS); and (c) Distant metastasis-free survival (DMFS). dMMR, defective mismatch repair; pMMR, proficient DNA mismatch repair.

**Supplementary Figure 2:** Association between neoadjuvant treatment and survival in patients with proficient DNA mismatch repair (pMMR). (a) Disease-free survival (DFS), (b) Local recurrence-free survival (LRFS), and (c) Distant metastasis-free survival (DMFS) in patients with pMMR tumor by neoadjuvant radiotherapy. NCT, neoadjuvant chemotherapy; NCRT, neoadjuvant chemoradiotherapy; HR, hazard ratio; CI, confidential interval.

**Supplementary Figure 3:** Cumulative incidence curves of events to show the probability of each competing event in the entire cohort (a, b) and according to dMMR (c, d), pMMR (e, f) and pMMR with stage III disease (g, h). The cumulative incidences of each competing event were presented and the differences between groups were calculated using Gray’s test. The red line represents local recurrence incidence and the black line represents the incidence of competing non- local recurrence associated death. The blue line represents distant metastasis incidence and the green line represents the incidence of competing non- distant metastasis associated death. LR= local recurrence; DM= distant metastasis; dMMR= deficient mismatch repair; pMMR= proficient mismatch repair; NCRT= neoadjuvant chemoradiotherapy; NCT= neoadjuvant chemotherapy

** Supplementary Figure 4:** Local recurrence-free survival (LRFS) in patients with stage II disease and proficient mismatch repair (pMMR) tumors by T stage (a) with neoadjuvant chemotherapy (NCT) alone and (b) neoadjuvant chemoradiotherapy (NCRT).

**Supplementary Figure 5:** Association between neoadjuvant treatment and survival according to mismatch repair (MMR) status in the remaining data excluding pCR. (a) Disease-free survival (DFS), and (b) Distant metastasis-free survival (DMFS) in the remaining data, patients with defective DNA mismatch repair (dMMR) by neoadjuvant radiotherapy. (c) DFS, and (d) LRFS in the remaining data, patients with stage III disease and proficient MMR (pMMR) tumors by neoadjuvant radiotherapy. NCT, neoadjuvant chemotherapy; NCRT, neoadjuvant chemoradiotherapy; HR, hazard ratio; CI, confidential interval.

**Supplementary Figure 6:** Local recurrence-free survival (LRFS) in the remaining data, patients with stage II disease and pMMR tumors by T stage (a) neoadjuvant chemotherapy (NCT) alone and (b) with neoadjuvant chemoradiotherapy (NCRT).

**Patient 3:**

A male patient, 35y, with dMMR tumor was diagnosed with poor differentiate rectal adenoma carcinoma invading peritoneal reflection (cT4a-bN1bM0) in January 2018 (Supplementary Figure 7a). After 1 cycle of XELOX and 2 cycles of FOLFOXIRI, pelvic MRI indicated progression of disease with T4bN2 (Supplementary Figure 7b). Then he received chemoradiotherapy with 3 cycles of FOLFOX. The radiation prescription is 45Gy for clinical target volume. After chemoradiotherapy, he had progression of disease with clinical stage cT4BN2M1 with lung and retroperitoneal lymph node metastases (Supplementary Figure 7c). Patient decided to receive immunotherapy with anti-PD-1 drug after the discussion with the experts in multidiscipline treatment group. After 16 cycles of immunotherapy, there was no high metabolic lesions in primary tumor and lung metastases and retroperitoneal lymph nodes shrinked a lot from the examination of PET/CT in August 20, 2019 and now he still received immunotherapy (Supplementary Figure 8).

**Supplementary Figure 7.** Magnetic resolution imaging of primary rectal carcinoma before treatment (a), after 3 cycles of chemotherapy (b), after chemoradiotherapy (c) and after 15 cycles of immunotherapy (d).


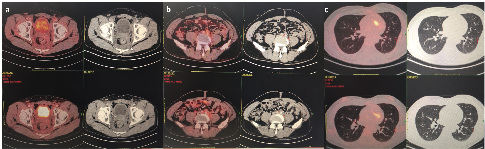


**Supplementary Figure 8.** PET/CT imaging of pelvic disease (a), retroperitoneal lymph nodes (b) and lung metastases (c).
